# Burst Strength of Tubing and Casing Based on Twin Shear Unified Strength Theory

**DOI:** 10.1371/journal.pone.0111426

**Published:** 2014-11-14

**Authors:** Yuanhua Lin, Kuanhai Deng, Yongxing Sun, Dezhi Zeng, Wanying Liu, Xiangwei Kong, Ambrish Singh

**Affiliations:** 1 State Key Laboratory of Oil and Gas Reservoir Geology and Exploitation (Southwest Petroleum University), Chengdu, Sichuan, 610500, China; 2 CNPC Key Lab for Tubular Goods Engineering (Southwest Petroleum University), Chengdu, Sichuan, 610500, China; 3 School of Material Science and Engineering (Sichuan University), Chengdu, Sichuan, 610000, China; Institute for Materials Science, Germany

## Abstract

The internal pressure strength of tubing and casing often cannot satisfy the design requirements in high pressure, high temperature and high H_2_S gas wells. Also, the practical safety coefficient of some wells is lower than the design standard according to the current API 5C3 standard, which brings some perplexity to the design. The ISO 10400: 2007 provides the model which can calculate the burst strength of tubing and casing better than API 5C3 standard, but the calculation accuracy is not desirable because about 50 percent predictive values are remarkably higher than real burst values. So, for the sake of improving strength design of tubing and casing, this paper deduces the plastic limit pressure of tubing and casing under internal pressure by applying the twin shear unified strength theory. According to the research of the influence rule of yield-to-tensile strength ratio and mechanical properties on the burst strength of tubing and casing, the more precise calculation model of tubing-casing's burst strength has been established with material hardening and intermediate principal stress. Numerical and experimental comparisons show that the new burst strength model is much closer to the real burst values than that of other models. The research results provide an important reference to optimize the tubing and casing design of deep and ultra-deep wells.

## Introduction

In “three-high” (high pressure, high temperature, and high H_2_S) gas wells, the service environment of tubing and casing becomes more complicated and harsh, and the internal pressure applied to tubing and casing becomes higher. Hence, the tubing and casing must have adequate burst strength to withstand the internal pressure without deformation. Furthermore, gas channeling and overflow may occur in case of burst of tubing and casing in “three-high” gas wells, which results in serious consequences [1]. Many scholars have done studies on strength of tubing and casing as the tubing and casing play an important role in the process of wellbore integrity, and many new methods and models (such as yield failure model, plastic collapse failure model and crack propagation model) which were used to analyze the strength of tubing and casing have been created [2]–[5]. But most of them only can predict the burst strength of thin-walled high-strength pipeline used to transport crude oil above the ground [6]–[7] instead of thick-walled tubing and casing underground.

However, the study on the API 5C3 strength model has found that the model can only predict the minimum internal pressure strength of tubing and casing. The basic failure criterion of API 5C3 depends on the initial yield of inner wall surface under the internal pressure, yet the casing has the ability of seal integrity and structure integrity in this situation. So, many tubular products were wasted in conventional wells and the tubing and casing strength often cannot satisfy the design requirement in rigorous wells (such as “three-high” gas wells) according to the current API 5C3 standard [8].

In addition, the initial yield in inner wall surface can't be presented in test or practical field. On the contrary, burst can truly reflect the loss of seal integrity in tubing and casing. Accordingly, ISO 10400:2007 [9] has proposed burst strength (ductile rupture strength) of tubing and casing and offered the model to calculate the burst strength of pipe (tubing and casing) under internal pressure. Compared with API 5C3 standard, the ISO burst strength model can predict the burst strength of tubing and casing according to the minimum wall thickness much better [10]–[12] and improve the design of tubing and casing strength to some extent. But, the study on the ISO strength model has found that its calculation precision is not desirable since approximately 50 percent predictive values are remarkably higher than real burst values [13]–[14], which goes against the design of tubing and casing strength and the improvement of upper limit of safety factor in the well Long-gang-001-1, Long-gang-001-2, Long-gang-13, Pu-guang-204-2H in Sichuan and Chongqing gas fields and the well Yingshen-1 in Tarim oilfield as well as the well TK1127 in Tahe oilfield in Xinjiang.

Moreover, in oil and gas industry, the tubing is the key component for transporting crude oil and gas, and the casing plays an important role in protecting and reinforcing borehole, isolating oil, gas and water during drilling and production. As a result, in order to improve the strength design of tubing and casing, on the basis of my work group results [13]–[14], based on the twin shear unified strength theory and twin shear stress yield criterion [15]–[16], the model that can calculate the burst strength of tubing-casing has been presented under capped-end conditions in this paper. Lots of numerical and experimental comparisons show that the prediction accuracy of “new burst strength model” is higher than that of ISO, and it can meet the requirement of engineering design of deep and ultra-deep wells.

## Mathematical Modeling

### API 5C3 internal pressure strength model

The model, which can calculate the minimum internal pressure strength of tubing and casing, has been presented by API Bulletin 5C3 [8] based on the yield design criterion by the following expression:
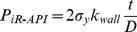
(1)where D is the outer diameter of pipe, mm; σ_y_ is the yield strength of pipe, MPa; and k_wall_ is the factor to account for the specified tolerance of the pipe wall. For example, for a tolerance of minus 12.5%, k_wall_ = 0.875; P_iR-API_ is the internal pressure strength calculated by Eq. (1), MPa; and t is the specified pipe wall thickness, mm.

For the API 5C3 strength model, the basic principle of yield caused by internal pressure is that initial yield in inner surface of pipe is failure, yet without loss of pressure/seal integrity. Initial yield in inner surface can't be presented in test or practical field. However, the pipe burst can truly reflect the loss of pressure/seal integrity in casing.

### ISO burst strength model under capped-end conditions

The yield design criterion can only meet the minimum design standard, and the API 5C3 model could not predict accurately the maximum load of tubing and casing. As a result, the ISO burst strength model under capped-end conditions has been proposed by Klever and Stewart [17]–[18]:
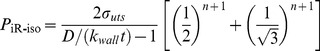
(2)where n is the dimensionless hardening exponent used to obtain a curve fit of the true stress-strain curve derived from the uniaxial tensile test (see ISO 10400: 2004 [9]), σ_uts_ is the tensile strength of specimen, MPa; and P_iR-iso_ is the burst strength calculated by Eq. (2), MPa.

The research on the ISO burst strength model has found that it can better predict the burst strength of tubing and casing by using the minimum wall thickness of pipe than API 5C3 [10]–[12]. However, it can be observed from the 106 groups of test data provided by three industry sources [9] that 50 percent predictive values of ISO burst strength model are remarkably higher than real burst values, which points against the design of tubing and casing strength and the improvement of upper limit of safety factor. Hence, the current ISO burst strength model cannot be used to predict precisely the burst strength of tubing/casing, which is not the purpose to propose the ISO burst strength model for the joint API/ISO work group ISOTC67 SC5 WG2b.

### New burst strength model of tubing and casing

#### (1) Twin shear unified strength theory

The twin unified strength theory can be transformed into many strength criterions (such as Tresca yield criterion, Von-Mises yield criterion and twin shear stress yield criterion) and some new criterions according to specific conditions [15]–[16], and it could be used to analyze limit load of various materials because the effects of intermediate principal stress and tensile/pressure ratio (the ratio of tensile strength to compressive strength) of material on the limit load have been taken into account. The mathematical expression of twin shear unified strength theory can be given as follows:
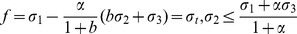
(1)

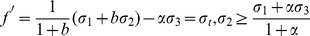
(2)where
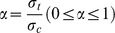


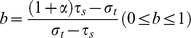
where σ_1_, σ_2_ and σ_3_ are the first, second/intermediate and third principal stress respectively, MPa; α is the tensile/pressure ratio; σ_t_ is the tensile strength, MPa; σ_c_ is the compressive strength, MPa; τ_s_ is the shear strength, MPa; b is the influence coefficient which reflects the effect of second/intermediate principal stress on the material failure.

#### (2) Elastic limit analysis of tubing and casing under internal pressure

**Figure 1:**
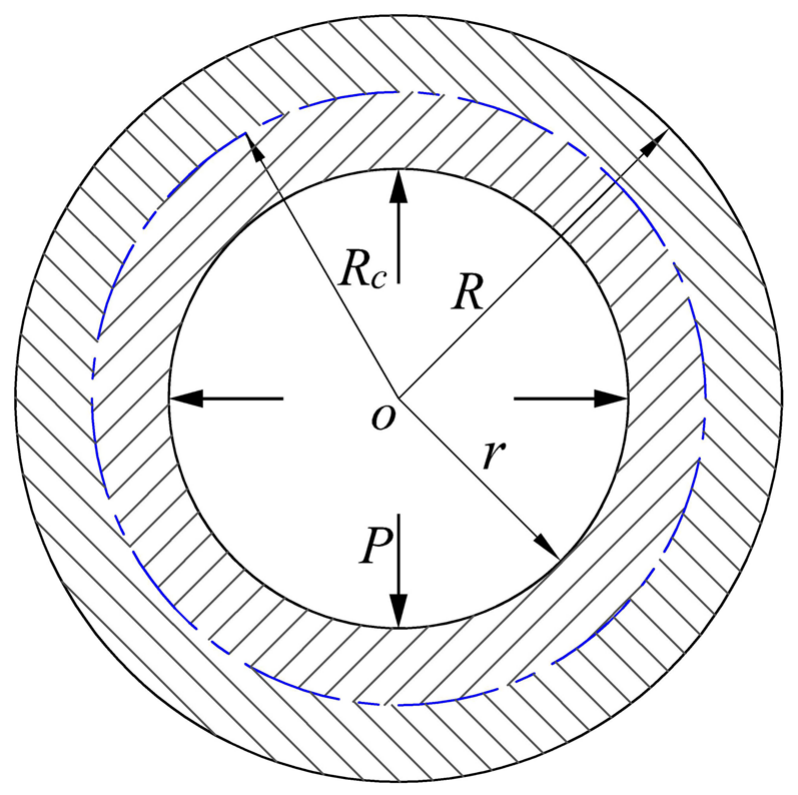
Mechanical model of pipe.

The stress components (radial stress σ_ρ_ and circumferential stress σ_θ_) of the tubing and casing increase with the increase of internal pressure P, as shown in **Figure 1**. The tubing and casing will be in the elastic limit state, when the inner wall yields firstly. Assume that the tubing and casing is a long thick-walled cylinder. In **Figure 1**, R is the outer radius; r is inner radius; P is the internal pressure at the inner surface of pipe. The long thick-walled cylinder is in the elastic state, when the internal pressure (P) is lower. According to the Lame formula [19], the following stress components can be obtained:
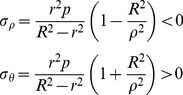
(3)


The mechanical analysis of long thick-walled cylinder under internal pressure is one of the axisymmetric plane strain problem. According to the study on the plane strain problem [20]–[21], the intermediate principal stress (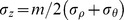, where m = 1) can be obtained in the elastic limit state of pipe. By the Eq. (3), Eq. (4) can be given:
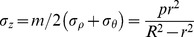
(4)


Based on the stress state of thick-walled cylinder, it could be known that the first, second and third principal stress (σ_1_, σ_2_ and σ_3_) are equal to σ_θ_, σ_z_ and σ_ρ_ respectively due to 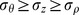. So the intermediate principal stress (σ_2_) is smaller than 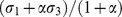 because the tensile/pressure ratio (α) is less than 1.0. Substituting Eq. (3) and Eq. (4) into Eq. (1) gives:
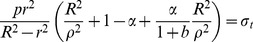
(5a)


To analyze the plastic limit load of thick-walled cylinder conveniently, the Eq. (5a) is simplified into Eq. (5b).
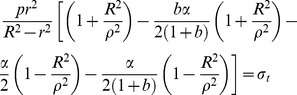
(5b)


The inner wall of thick-walled cylinder yields firstly, when the internal pressure increases to the yield strength of the material. By the Eq. (5), the elastic limit load (Py) can be obtained:
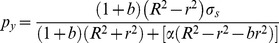
(6)where the P_y_ is the elastic limit load (internal pressure strength), MPa; σ_s_ is the yield strength of tubing and casing, MPa.

#### (3) Plastic limit analysis of tubing and casing under internal pressure

The plastic region will be formed near the inner wall of thick-walled cylinder, when internal pressure (P) is larger than elastic limit load (P_y_). Assume that R_c_ is the radius of interface between elastic region and plastic region, mm, as shown in **Figure 1**. The plastic region will extend from inner surface to outer surface with the increase of internal pressure (P), which will result in the range of 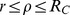
becoming plastic region and the range of 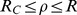 becoming elastic region, as shown in **Figure 1**.

**Figure 2:**
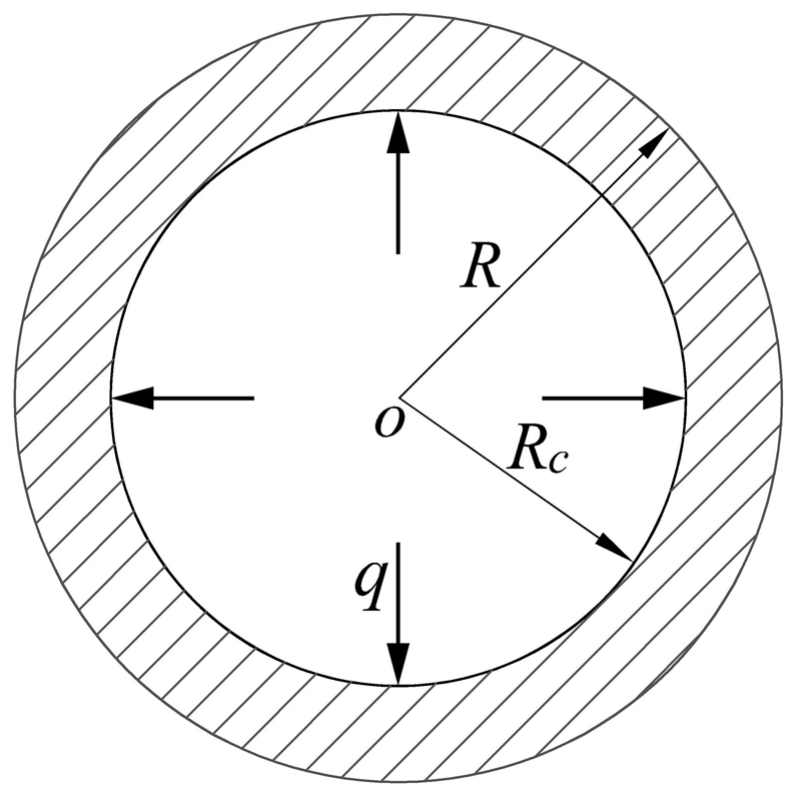
Elastic region (Outer cylinder).

**Figure 3:**
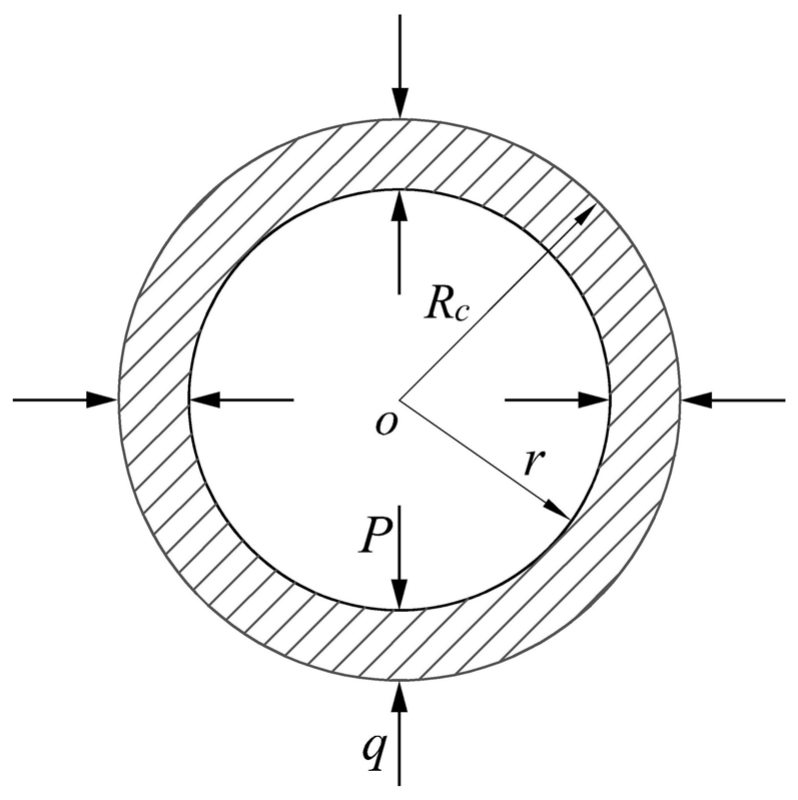
Plastic region (Inner cylinder).

The interface between the elastic region and plastic region is cylinder surface due to the axial symmetry of stress components (σ_θ_ and σ_r_). Assume that the elastic region is an outer cylinder, as shown in **Figure 2**, and the plastic region is an inner cylinder, as shown in **Figure 3**. Therefore, the elastic and plastic regions are analyzed based on the principle of long thick-walled cylinder. According to axial symmetry, the radial pressure (q) is applied to outer wall of inner cylinder and inner wall of outer cylinder respectively, as shown in **Figure 2** and **Figure 3**.

In plastic region (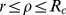), as shown in **Figure 3**, based on the equilibrium equation of plastic mechanics [19], the Eq. (7) can be obtained:
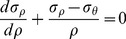
(7)


By the Eq. (3) and Eq. (5b), the Eq. (8) can be obtained:
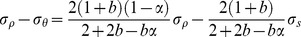
(8)


Substituting Eq. (8) and boundary conditions (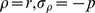) into Eq. (7) gives:
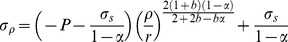
(9a)

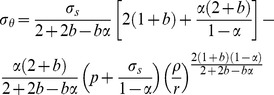
(9b)


In elastic region (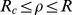), as shown in **Figure 2**, according to the lame formula, the stress components can be obtained:
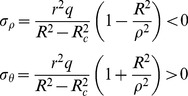
(10)where
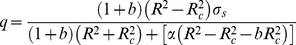
(11)


According to continuity of the radial stress (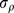), the radial stress of elastic region is equal to plastic region at the interface (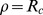). As a result, by the Eq. (9a), Eq. (10) and Eq. (11), the Eq. (12) can be obtained:
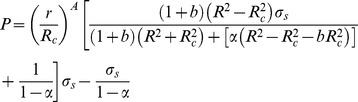
(12)where
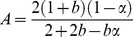



The plastic region increases with the increase of internal pressure (P). The whole wall of thick-walled cylinder is in plastic state, when the radius of interface (R_c_) is equal to the outer radius (R) of thick-walled cylinder. According to the Eq. (12), the plastic limit load (P_p_) can be obtained:
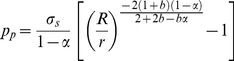
(13)


Based on the twin shear yield criterion, by the Eq. (13), the burst strength model can be obtained:
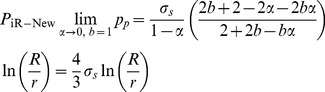
(14)


The Eq. (14) used to calculate burst strength of real tubing and casing based on the yield strength (σ_s_) of tubing and casing is not reasonable and needs to be further improved because the real tubing and casing will undergo the hardening stage and large plastic deformation from inner wall yield to whole wall yield. Hence, the yield strength (σ_s_) of tubing and casing has been replaced by flow stress (σ_f_) to calculate the burst strength accurately and reasonably in this paper. The Klever's research results [17] also demonstrated that the flow stress (σ_f_) which can deal with the effect of hardening and larger plastic deformation on the burst strength of tubing and casing ranged between the yield strength and tensile strength. In addition, for the different metal pipe, some empirical formulas of flow stress have been proposed by some scholars [22]–[24], as follows:
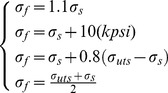
(15)


The study on the Eq. (15) has indicated that the yield-to-tensile strength ratio (the ratio of yield strength to the tensile strength) has a great effect on the burst strength of tubing and casing. Especially, for the lower the yield-to-tensile strength ratio and, smaller the yield-to-tensile strength ratio, the capacity for plastic deformation is higher [25], which is in agreement with my work group results [26]. So, on the basis of results of our work group [13]–[14], a new burst strength model (Eq. (16)) for predicting the burst strength of tubing and casing under capped-end conditions is presented with consideration of the yield-to-tensile strength ratio, manufacturing imperfection (including the ovality and eccentricity of pipe), crack defects (the crack depth of pipe), material hardening (the phenomenon that the material strength and hardness increase in the process of plastic deformation [27]) etc.:
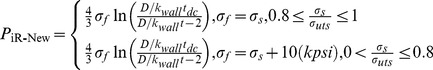
(16)where t_dc_ = t-ak_a_, a is the crack depth that should be less than 5% of the wall thickness, mm; k_a_ is the burst strength factor, having the numerical value 1, 0 for quenched and tempered (Martensitic structure) or 13Cr products and 2, 0 for as rolled and normalized products based on available test data; and the default value set to 2, 0 where the value has not been measured [9].

The new burst strength model (Eq. (16)) could predict burst strength of tubing and casing accurately and reasonably, which will make great improvements in the tubing and casing design of deep and ultra-deep wells on the basis of material safety which was guaranteed.

## Results and Discussion

**Figure 4:**
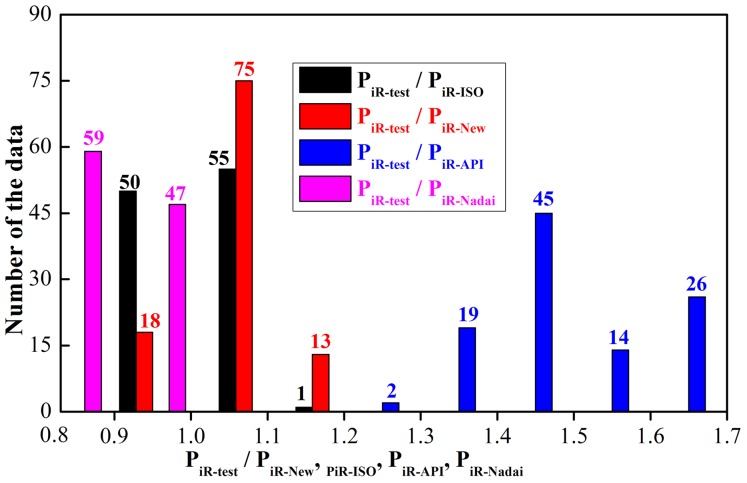
Ratio of experiment data to the calculation results of burst strength.

In order to validate the accuracy and reliability of the new burst strength model (Eq. (16)), the calculation results of the new model have been compared with the calculation results of the API model [8], ISO model [9], Nadai model [28] and the 106 groups of experiment data (P_iR-test_) donated from three industry sources under capped-end conditions due to the high internal pressure [9]. The comparisons of calculation results and experiment data are shown in **Figure 4**. In **Figure 4**, the x-coordinate is the ratio of experiment data (P_iR-test_) to calculation results of model; the y-coordinate is number of the data; P_iR-ISO_ is the burst strength calculated by the ISO model; P_iR-New_ is the burst strength calculated by the new model; P_iR-API_ is the burst strength calculated by the API model; P_iR-Nadai_ is the burst strength calculated by the Nadai model.

From **Figure 4**, the following may be noted.

For the 106 groups of ratio of P_iR-test_/P_iR-New_, 75 groups (70.8%) fall in the closed interval [1.0**–**1.1]; 13 groups (12.3%) fall in the closed interval [1.1**–**1.2]; and only 18 groups (16.9%) fall in the closed interval [0.9**–**1.0]. For the 106 groups of ratio P_iR-test_/P_iR-ISO_, 50 groups (about 50%) of calculation are higher than the test data. For the 106 groups of ratio P_iR-test_/P_iR-API_, all the ratios are considerably larger than 1.2, which demonstrates all the calculations are considerably less than test data. For the 106 groups of ratio P_iR-test_/P_iR-Nadai_, all the ratios are considerably smaller than 1.0, and 55.6 percent of the calculations are 20 percent larger than test data.

**Figure 5:**
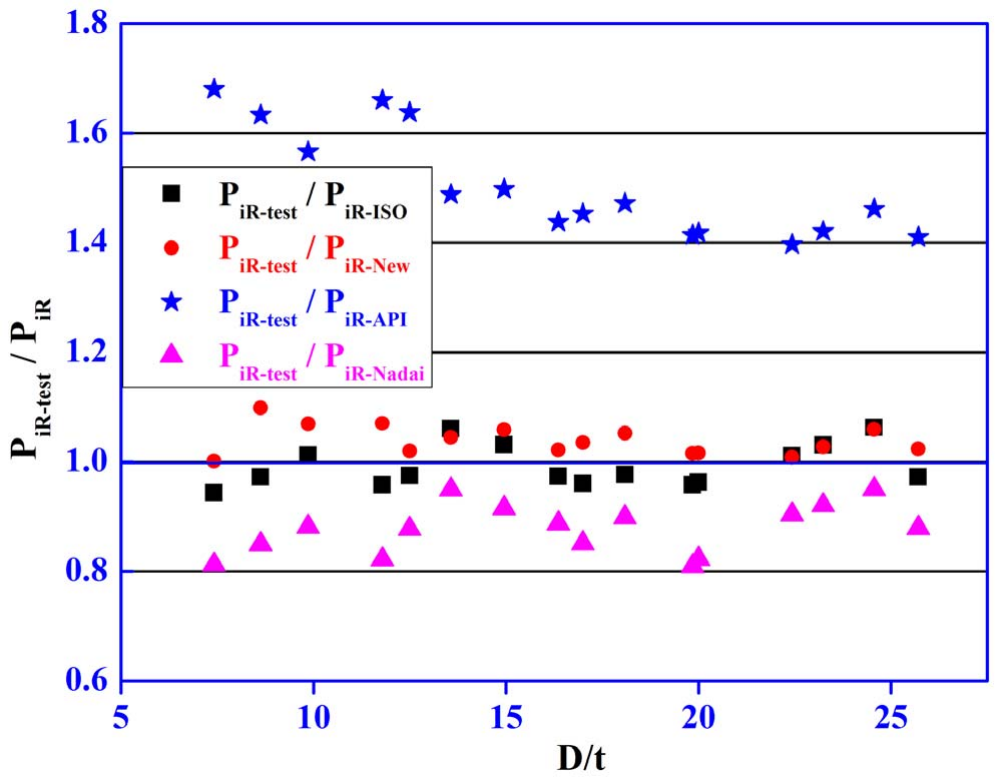
Relationship between the ratio of test data to calculations and the radius-thickness ratio (D/t).

To validate the accuracy of the new model, the calculations of burst strength for the 16 kinds of frequently-used casing (7<D/t<26) have been presented in Table 1. In general, it can be observed from Table 1 that the calculations of model are P_iR-API_<P_iR-New_<P_iR-ISO_<P_iR-Nadai_. It can be seen from **Figure 5** that P_iR-API_ is considerably less than the test data; P_iR-Nadai_ is considerably larger than the test data; P_iR-ISO_ is close to the test data, but its calculations is almost larger than the test data, which makes against the casing design and improvement of upper limit of safety coefficient. However, P_iR-New_ is quiet closer to the test data and its calculations are only slightly less than the test data (to meet the requirement of engineering design, the calculations must be less than real values, but not 10 percent less than real values), which is beneficial to casing design of deep and ultra-deep wells and improvement of upper limit of safety coefficient on the basis of material safety which was guaranteed. So the new model is more accurate and reasonable than other models.

**Table 1:** Comparison of burst strength calculation results with test data.

No.	D/t	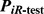 MPa	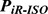 MPa	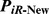 MPa	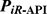 MPa	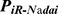 MPa	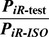	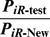	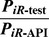	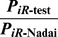
1	7.42	175.5	186.0	175.3	102.0	216.1	0.944	1.001	1.680	0.812
2	8.63	163.3	168.0	148.7	100.0	192.2	0.972	1.099	1.633	0.850
3	9.86	151.2	149.4	141.4	96.6	171.5	1.012	1.069	1.566	0.882
4	11.79	88.9	92.9	83.1	48.8	108.2	0.958	1.070	1.660	0.822
5	12.5	179.1	183.8	175.7	141.2	204.1	0.975	1.020	1.638	0.878
6	13.57	173.8	163.9	166.4	116.8	183.1	1.060	1.045	1.488	0.949
7	14.95	136.1	132.0	128.6	90.9	148.7	1.031	1.059	1.498	0.915
8	16.36	143.0	146.8	139.9	99.5	161.1	0.974	1.022	1.437	0.887
9	17	76.8	80.0	74.2	52.9	90.2	0.961	1.035	1.453	0.851
10	18.09	142.7	146.1	135.7	97.0	158.8	0.977	1.052	1.472	0.899
11	19.85	83.5	87.2	82.2	59.1	103.1	0.957	1.015	1.414	0.809
12	20	83.1	86.3	81.8	58.6	101.0	0.963	1.016	1.418	0.823
13	22.43	93.4	92.5	92.3	66.9	103.4	1.011	1.010	1.397	0.904
14	23.24	91.7	89.3	89.2	64.6	99.6	1.028	1.028	1.420	0.921
15	24.56	89.3	84.5	84.3	61.1	94.0	1.060	1.061	1.461	0.950
16	25.71	80.6	82.9	78.7	57.2	91.6	0.972	1.023	1.410	0.879

The new model is more accurate and beneficial to design burst strength of the tubing and casing and improves the upper limit of safety coefficient than other models according to the numerical and experimental comparisons. The design principal of API model is so conservative that the internal pressure strength has a large margin when the inner wall of pipe yields. The Nadai model calculates the burst strength of pipe by using the tensile strength directly without consideration of material hardening so that the calculations are obviously larger than test. The calculations of ISO model is close to test data with consideration of material hardening, but it could overestimate the limit loads and give unsafe predictions for tubing and casing, which is detrimental to the design of real tubing and casing and the increase proves the upper limit of safety coefficient to some degree.

The study results could provide references for the study on the impact of tensile/pressure ratio (the ratio of tensile strength to compressive strength) [29] on the internal pressure strength. For three high gas wells, the study of my work group [30] shows the failure mode belongs to crack expansion instability, when the crack depth is more than 5% of the wall thickness. The tubing and casing should be designed by combining the theory of fracture mechanics and strength theory.

## Conclusions

For the sake of improving the calculation and design accuracy of API and ISO standard, based on the twin shear unified strength theory and twin shear stress yield criterion, a new burst strength equation has been presented with due consideration of manufacturing imperfections, yield-to-tensile strength ratio, crack defects, material hardening and intermediate principal stress in its calculation so that the new model can better reflect actual mechanical properties of the tubing and casing and improve the burst strength calculation accuracy.

Numerical and experimental comparisons show that the calculation accuracy of Eq. (16) is better than that of API and ISO. Hence, the Eq. (16) can be used as the lower bound design equation, which will make great improvements in “three high” gas wells tubing and casing designs, rather than just using API bulletin 5C3 or ISO because not only does it avoid the material loss but also guarantees the safety and reliability of material.
